# Prevalence and intensity of genito-urinary schistosomiasis and associated risk factors among junior high school students in two local government areas around Zobe Dam in Katsina State, Nigeria

**DOI:** 10.1186/s13071-016-1672-5

**Published:** 2016-07-07

**Authors:** Tolulope Ebenezer Atalabi, Umar Lawal, Simeon Johnson Ipinlaye

**Affiliations:** Department of Biological Sciences, Faculty of Science, Federal University, Dutsin-Ma, P.M.B. 5001, Dutsin-Ma, Katsina State Nigeria; Federal College of Education, P.M.B. 3045, Gwale Local Government, Kano State Nigeria

**Keywords:** Genito-urinary, Schistosomiasis, Risk factors, Prevalence, Intensity, Dutsin-Ma, Safana, Nigeria

## Abstract

**Background:**

The national burden of genito-urinary schistosomiasis in Nigeria has been expressed by an estimate of 101.3 million people at risk with an alarming figure of 29 million infected. Report obtained from respondents about Praziquantel® distribution and the obviously prevalent haematuria without any control programme in place justified the need for data on the prevalence, intensity of infection and associated risk factors which were the objectives this cross-sectional survey sought to address.

**Methods:**

A total of 718 students aged 10–23 years from the study area were surveyed between May and August, 2015. Data on socio-demographic and risk factors were obtained using structured questionnaires. Clean universal bottles with corresponding labels were offered for sample collection between 10:00 am and 13:30 pm. Centrifuged samples were microscopically examined and intensity of infection was recorded per 10 ml of each sample.

**Results:**

Prevalence of genito-urinary schistosomiasis was 22.7 % with a mean intensity of 25.05 (± standard deviation, ± 71.51) eggs/10 ml of urine. Higher prevalence (19.5 %) and mean intensity (28.7 eggs/10 ml of urine) was recorded among boys. Sex (*χ*^2^ = 77.065, *P* < 0.0001), age group 16–18 (*χ*^2^ = 5.396, *P* = 0.0202), altitude (*χ*^2^ = 8.083, *P* = 0.0045), unwholesome water sources (*χ*^2^ = 27.148, *P* < 0.0001), human recreational activities (*χ*^2^ = 122.437, *P* < 0.0001), mothers’ occupation (*χ*^2^ = 6.359, *P* = 0.0117), farming (*χ*^2^ = 6.201, *P* = 0.0128) and other brown collar jobs (*χ*^2^ = 4.842, *P* = 0.0278) in fathers’ occupational category were found to be significantly associated with urogenital schistosomiasis. Boys were seven times more likely to be infected compared to girls [AOR (95 % CI): 7.3 (4.26–12.4)]. Age group 16–18 years was four times more likely to be infected compared to age group 10–12 years [AOR (95 % CI): 4.43 (2.62–7.49)]. Similarly, respondents with farming as fathers’ occupation were twice more likely to be infected than those whose fathers were civil servants [AOR (95 % CI): 2.08 (1.2–3.59)].

**Conclusions:**

Dutsin-Ma and Safana were classified as moderate-risk Local Government Areas (LGA). Sex, altitude, unwholesome water sources and mothers’ occupation were identified as the determining epidemiological factors in the prevalence of the disease. Sustainable chemotherapeutic intervention with Praziquantel®, good network of treated pipe-borne water, health education and waste disposal facilities are highly recommended to reduce its prevalence below the threshold of public health significance.

## Background

Globally, genito-urinary schistosomiasis remains a significant public health burden by consistently ranking a second position to malaria in terms of its socio-economic and public health importance [1]. It is closely associated with squalor-ravaged rural dwellers of tropical and sub-tropical regions of the world [2]. With the common knowledge that many endemic loci remain under-researched, researchers have underestimated the continental burden of the disease by reporting that 436 million and 112 million people, respectively, were at risk and infected in sub-Saharan Africa [3] by the cercariae of *Schistosoma haematobium*.

Various species of freshwater snail belonging to the genus *Bulinus* act as intermediate hosts of this aetiological agent. Disease transmission is further aided by various human water contact activities for recreational and domestic purposes [4]. Early symptoms of the disease include haematuria, anaemia and retardation of growth and development in children. At advanced stage, it results in cancer of the bladder, hepatic and renal malfunctions. In Africa, the morbidity of schistosomiases culminates in the mortality rate of an estimated 280,000 people per year [5].

In Nigeria, the burden of schistosomiasis has been expressed by an estimate of 101.3 million people who were at risk with an alarming figure of 29 million infected. The latter depicts Nigeria as the most endemic country in sub-Saharan Africa for schistosomiasis followed by Tanzania where 19 million people were infected [6].

The report obtained from respondents about Praziquantel® distribution as well as the obviously prevalent haematuria without any control programme in place justified the need for data on the prevalence, intensity of infection and associated risk factors which were the objectives this cross-sectional survey sought to address.

## Methods

### Study area

The survey was carried out in Safana (12°24.5′N, 7°24.4′E) and Dutsin-Ma (12°26′N, 7°29′E) Local Government Areas (LGAs) of Katsina State, Northwestern Nigeria. Covering a total area of 809 km^2^, both LGAs were inhabited by 353,450 people as at 2006 National Census [7]. Dutsin-Ma has a mean annual rainfall of 700 mm (May to September) and temperature range of 29–31 °C [8]. The ethnic groups comprise the Hausa and Fulani who are predominantly cereal crop farmers, animal rearers and traders. Zobe Dam, the major water body, although located in the Western outskirt of the latter, is however, close to the eastern part of the former and boosts green revolution with irrigation capacity of 8000 ha [9].

### Study design, duration and respondents

The cross-sectional study was carried out between May and August, 2015. It was designed to target a total number of 718 junior high school students from Darawa, Sokoto Rima, Kofa, Tsaskiyya, Kofa Fada and Safana communities.

Students in the age group 10–23 years voluntarily enrolled in the study. Meanwhile, student(s) who were severely ill, unwilling and those who either refused to return samples or supplied chemical in place of urine were not included in the survey.

A total of six schools was selected from the twenty junior high schools in both LGAs: Government Pilot Junior Secondary School (GPJSS), Safana; Government Junior Secondary School (GJSS), Tsaskiyya; Community Day Junior Secondary School (CDJSS), Safana; Government Pilot Junior Secondary School (GPJSS), Dutsin-Ma; Community Day Junior Secondary School (CDJSS), Dutsin-Ma and Government Junior Secondary School (GJSS), Dutsin-Ma. Sample size was allotted to each school and by extension, each class of participating students. Simple random sampling technique was employed to enrol 718 students from a total of about 4674 students in the selected schools.

### Sample collection and processing

Forms A, B and C representing school-based, individual-based questionnaires and urine analysis forms, respectively, were used to collect data during the survey. The school-based questionnaire was administered to each principal. Among others, this was used to collect data on students’ population, history of Praziquantel® distribution, report of haematuria and local languages for haematuria and *Bulinus* species. Individual-based questionnaire was employed to collect information on name, age, sex, water sources and water contact activities such as swimming and playing in shallow water. The urinalysis form was used to record urine parameters.

Clean, screw-capped and labelled plastic universal bottles (20 ml) were offered to the respondents who produced various quantities of terminal urine samples between the hours of 10:00 and 14:00 [10, 11] which coincided well with the circadian rhythm of egg excretion in *S. haematobium* [12]. About 5 ml of each urine sample was measured into test-tube and centrifuged at 2250 rpm for 1.5 min using Centurion Scientific Centrifuge (C2 series). The supernatant fluid was decanted off while the sediment (containing eggs) was examined under light microscope.

Adjustable micro-pipette (10–100 μl) was used to introduce 10 μl of each sample onto a clean, grease free glass slide and covered at an angle with a glass slip to avoid bubble formation. Microscopic examination of wet preparation for the eggs of *S. haematobium* was carried out using 10 × objective nose of Motic® Binocular Compound Light Microscope. Samples with eggs were recorded as positive while those without eggs were taken to be negative. For positive samples, egg counts were carried out and each average count was recorded as number of eggs per 10 ml of urine sample [13]. Intensity of infection was categorised into light (< 50 eggs/10 ml of urine) and heavy (≥ 50 eggs/10 ml of urine) infections [14].

### Quality assurance

Each universal bottle was assigned a serial number which corresponded to the number on each individual-based questionnaire. Urine samples collected were promptly analysed at the sites of collection with the data on each reagent strip recorded within 90 s. Where this was impossible, they were quickly transported to the Biology Laboratory of the Federal University, Dutsin-Ma. Noteworthy was the fact that accuracy and thoroughness might be compromised due to the stress of having to examine all the samples under the microscope. To circumvent this, a rapid means of screening out negative samples was adopted. Average counts of *S. haematobium* eggs were rather recorded. On few occasions where intensities were extremely light, averages of six or seven counts were recorded.

### Statistical analysis

Data obtained from the survey were entered into Microsoft Excel 2010 and analysed with SPSS version 15.0. Data summary was carried out using frequency and crosstabs sections of descriptive statistics. Associations among variables were determined using Chi-square test and bivariate and multivariate logistic regression. Statistical significance was considered at 95 % Confidence Level (CL) with a *P-*value of less than 0.05. Strength of association was measured by means of Odds Ratio (OR) at 95 % CI. Adjustment was made for OR by using haematuria.

## Results

A total number of 718 junior high school students were enrolled for the survey. The mean age (± standard deviation, SD) of the respondents was 13.96 ± 1.74 years. White collar and brown collar jobs were the major occupational categories of the respondents’ fathers (Table 1). Table 2 provides information on the sources of water for recreation and domestic uses among interviewees surveyed.

**Table 1:** Socio-demographic features of respondents from Safana and Dutsin-Ma LGAs

Variables	Frequency	Percentage	Cumulative percentage
Age (years)			
10–12	126	17.5	17.5
13–15	485	67.5	85.1
16–18	94	13.1	98.2
19–21	11	1.6	99.7
22–24	2	0.3	100
Sex			
Male	401	55.8	44.2
Female	317	44.2	100
Fathers’ occupation			
White collar jobs	249	34.7	34.7
Farming	115	16	50.7
Other brown collar jobs	351	48.9	99.6
Late	2	0.3	99.9
Retiree	1	0.1	100
Mothers’ occupation			
White collar jobs	76	10.6	10.6
Farming	2	0.3	10.9
Other brown collar jobs	390	54.3	65.2
Housewives	250	34.8	100
Total	718	100	100

**Table 2:** Frequency of risk factors associated with Genito-urinary schistosomiasis from Safana and Dutsin-Ma LGAs

Variables	Frequency	Percentage	Cumulative percentage
Swimming experience			
Yes	237	33	33
No	481	67	100
Total	718		
Playing in shallow water			
Yes	330	46	46
No	388	54	100
Total	718		
Itching experience			
Yes	140	19.5	19.5
No	578	80.5	100
Total	718		
Sources of water for drinking, cooking, washing & bathing			
Bore hole	463	30.89	30.89
Dam	174	11.61	42.5
Pond	120	8.01	50.51
River	53	3.54	54.05
Stream	198	13.21	67.26
Well	243	16.21	83.47
Tap	246	16.4	99.87
Sachet	2	0.13	100
Total	1499	100	

**Fig. 1:**
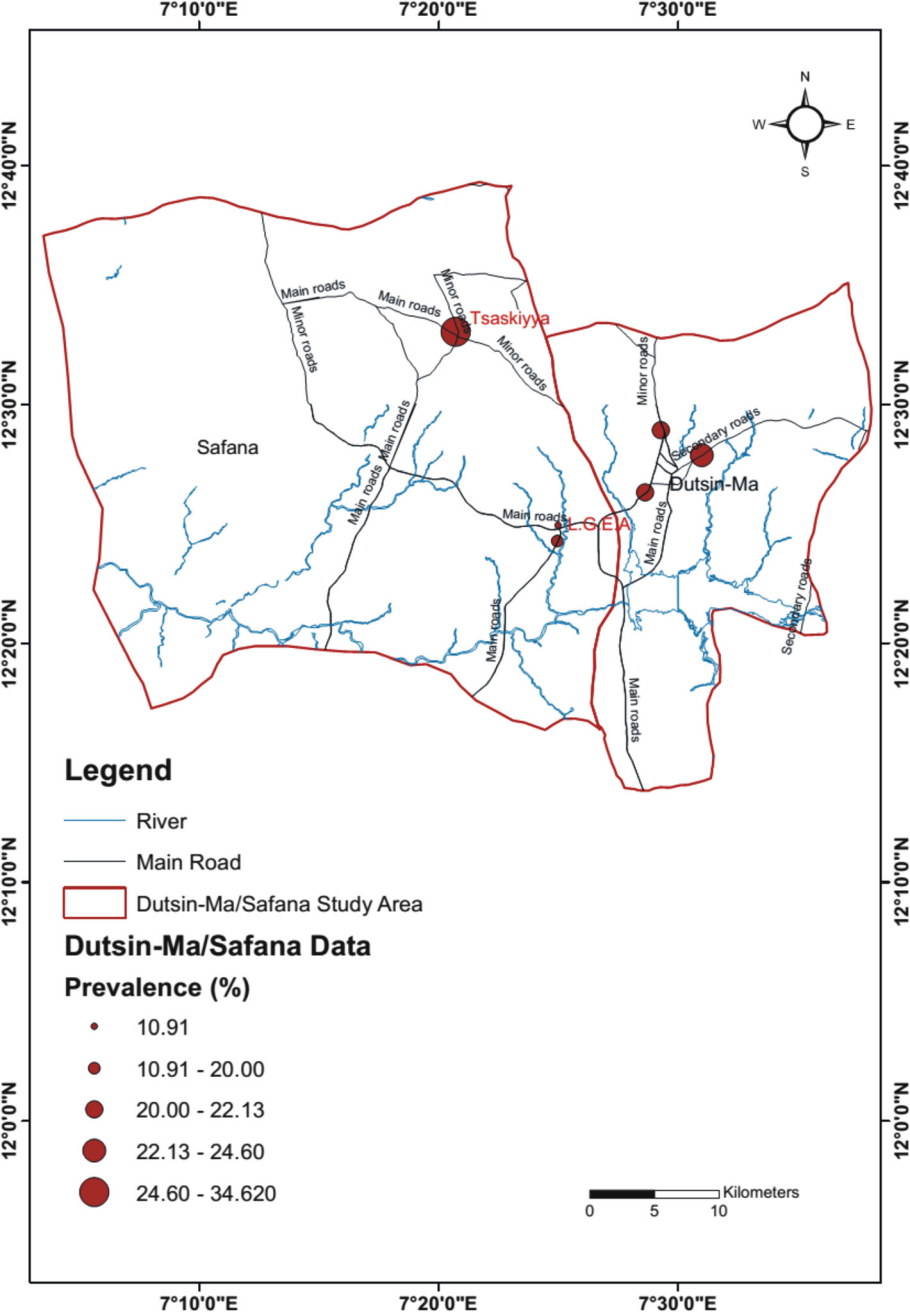
Map of the study area showing the prevalence of urogenital schistosomiasis by study location

**Fig. 2:**
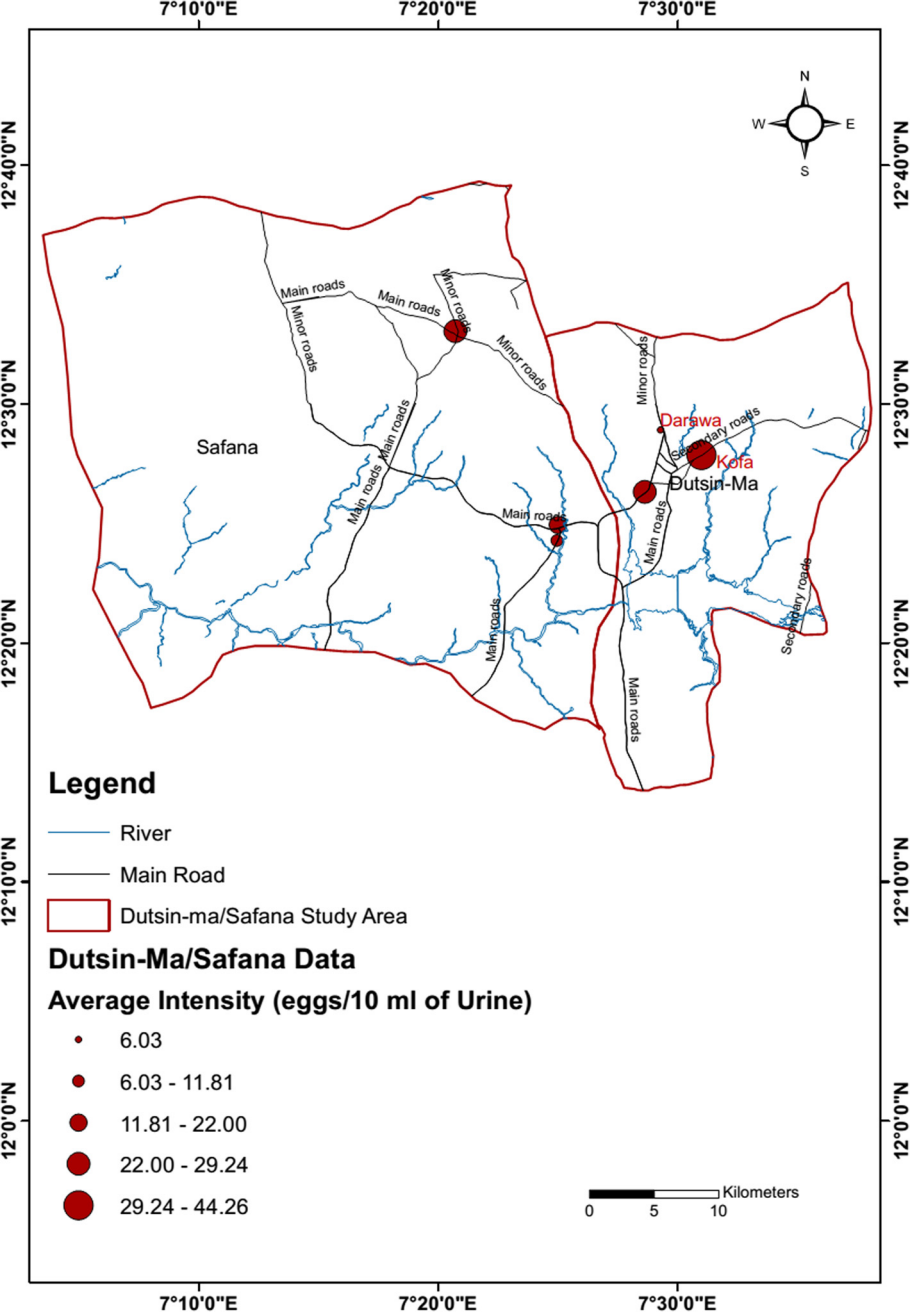
Map of the study area showing the mean intensity of urogenital schistosomiasis by study location

The highest (34.62 %) and lowest (10.91 %) prevalence values were recorded among participants from Tsaskiyya and Local Government Educational Authority (LGEA) communities in Safana, respectively (Fig. 1). Meanwhile, the highest and lowest values of mean intensity were recorded among respondents from Kofa (44.26 eggs/10 ml of urine) and Darawa (6.03 eggs/10 ml of urine) communities in Dutsin-Ma (Fig. 2).

**Fig. 3:**
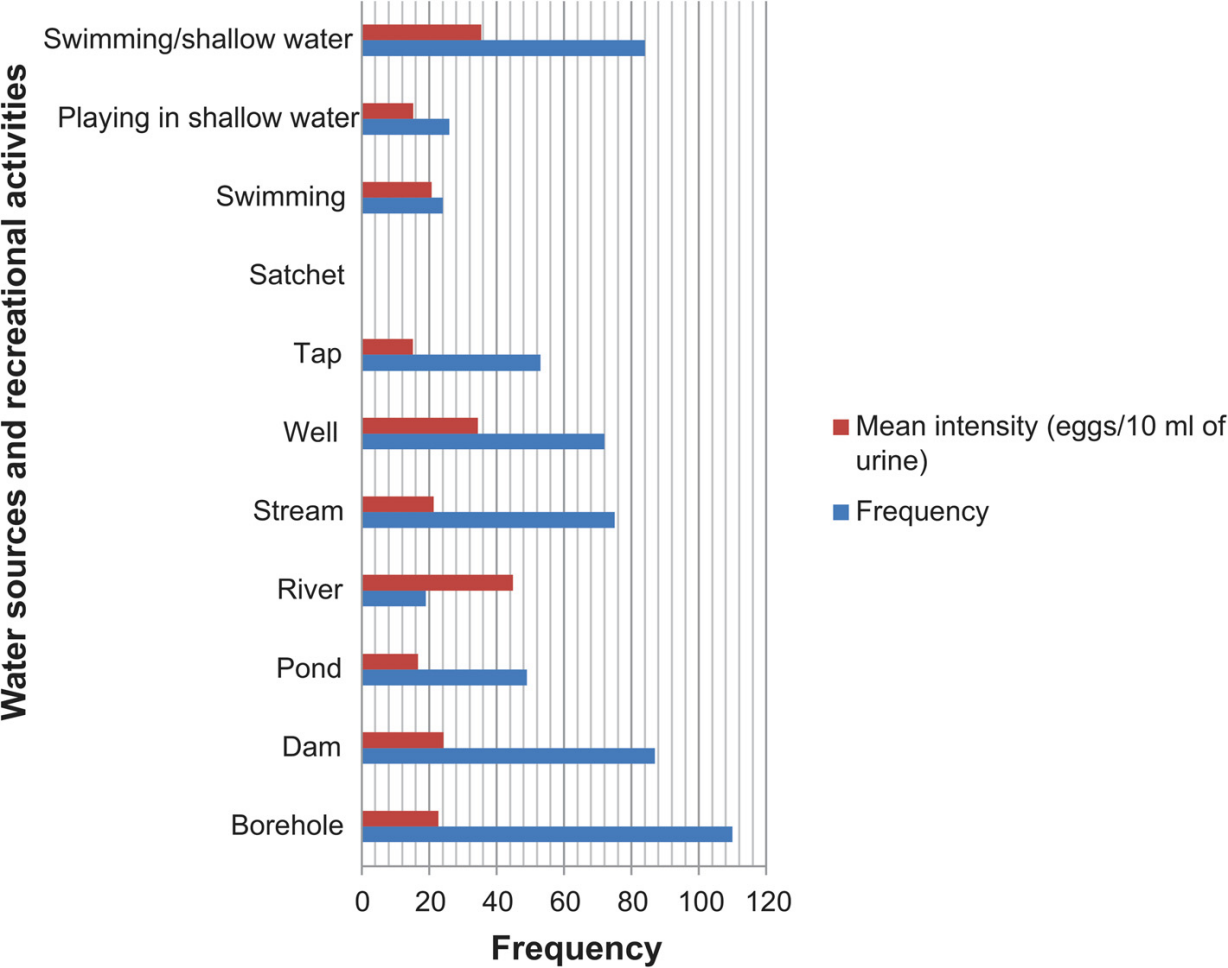
Water sources/recreation-related frequency and intensity of infection with *S. haematobium* cercariae in the study LGAs

The highest frequency of infection (50 %) was recorded among respondents who relied on dam water for domestic use, followed by 40.83 and 37.88 % recorded among users of ponds and streams, respectively. However, the mean intensity of infection was highest among users of rivers [44.89 (2.71–87.08) eggs/10 ml of urine] followed by wells [34.44 (10.09–58.79)] and dams [24.21 (13.77–34.64)] (Table 3 and Fig. 3).

**Table 3:** Frequency and intensity of genito-urinary schistosomiasis in relation to water sources for domestic and recreational uses

Water source	Number examined (%)	Number infected (%)	Odds ratio (95 % CI)	*P-*value	ECAM (95 % CI)
Borehole	463 (100)	110 (23.76)	1.13 (0.78–1.65)	0.5052	2497 [22.7 (14.28–31.12)]
Dam	174 (100)	87 (50)	3.63 (2.38–5.58)	< 0.0001	2106 [24.21 (13.77–34.64)]
Pond	120 (100)	49 (40.83)	2.51 (1.56–4.04)	0.0001	819 [16.71 (10.55–22.87)]
River	53 (100)	19 (35.85)	2.03 (1.06–3.84)	0.0274	853 [44.89 (2.71–87.08)]
Stream	198 (100)	75 (37.88)	2.22 (1.46–3.38)	0.0002	1602 [21.36 (9.95–32.77)]
Well	243 (100)	72 (29.63)	1.53 (1.02–2.32)	0.0406	2480 [34.44 (10.09–58.79)]
Tap	246 (100)	53 (21.54)	1.00 (0.65–1.54)	1.0000	802 [15.13 (7.32–22.95)]
Sachet	2 (100)	0	0	–	0
Recreational activities					
Swimming	237 (100)	24 (10.13)	1.32 (0.73–2.37)	0.3523	496 [20.67 (9.71–31.62)]
Playing in shallow water	330 (100)	26 (7.88)	1	–	397 [15.27 (1.45–29.09)]
Swimming & playing in shallow water	151 (100)	84 (55.63)	14.55 (8.78–24.64)	< 0.0001	2981 [35.49 (14.73–56.25)]

In the ECAM column, the raw egg counts are followed by the mean egg counts and the confidence intervals in parentheses

*Abbreviation*: *ECAM* Egg count arithmetic mean

In the use of water for recreation, respondents who combined playing in shallow water with swimming had the highest frequency (55.63 %) and mean intensity [35.49 (14.73–56.25)] of *S. haematobium* infection. Swimmers recorded a second highest frequency and mean intensity of 10.13 % and [20.67 (9.71–31.62) eggs/10 ml of urine], respectively (Table 3, Fig. 3).

In the bivariate analysis, dams [COR (95 % CI): 3.63 (2.38–5.58)], ponds [COR (95 % CI): 2.51 (1.56–4.04)], rivers [COR (95 % CI): 2.03 (1.06–3.84)], streams [COR (95 % CI): 2.22 (1.46–3.38)] and wells [COR (95 % CI): 1.53 (1.02–2.32)] were found to be significantly associated with the frequency of urogenital schistosomiasis.

In the recreation aspect, only swimming combined with playing in shallow water [COR (95 % CI): 14.55 (8.78–24.64)] was found to be significantly associated with the disease (Table 3).

Nevertheless, a weak linear relationship as well as a statistically significant association existed between egg counts of *S. haematobium* and swimming (*r* = 0.1752, *P* < 0.0001), playing in shallow water (*r* = 0.1179, *P* = 0.0016), fetching water from river (*r* = 0.08265, *P* = 0.0268). A statistically insignificant association (*P* = 0.2641) coupled with a weak linear relationship (*r* = 0.04173), however, existed between egg count and fetching water from stream.

In this study, the prevalence of genito-urinary schistosomiasis was 22.7 % with a mean (± SD) intensity of 25.05 ± 71.51) eggs/10 ml of urine. Of the 718 urine samples examined, 163 (22.7 %) tested positive for eggs of *S. haematobium*, with a total raw egg count (mean) of 4083 [25.05 (13.99–36.11)] (Table 4).

**Table 4:** Prevalence and intensity of genito-urinary schistosomiasis in relation to sex, age, LGAs, category of *S. haematobium* infection and parental occupation of respondents

Variables	Number examined	Number infected	Prevalence (%)	Raw egg count [Mean (95 % CI)]
Sex				
Boys	401	140	19.5	4018 [28.7 (15.9–41.49)]
Girls	317	23	3.2	65 [2.83 (1.71–3.95)]
*χ* ^*2*^ test			77.065	
*P-*value			< 0.0001	< 0.05
Age (years)				
10–12	126	22	3.06	636 [28.91 (5.72–52.09)]
13–15	485	107	14.9	2844 [26.58 (10.5–42.65)]
16–18	94	29	4.04	588 [20.28 (9.71–30.84)]
19–21	11	4	0.56	8 [2 (0.16–3.84)]
22–24	2	1	0.14	7 (7)
*χ* ^*2*^ test			0.642	–
*P-*value			0.4231	0.9981
Address				
Safana	320	73	10.17	1769 [24.23 (12.07–36.39)]
Dutsin-Ma	398	90	12.53	2314 [25.71 (8.05–43.37)]
*P-*value				1.064
Infection category				
Light (≤ 50 eggs/10 ml)	146	146	20.33	1543 [10.57 (8.48–12.66)]
Heavy (≥ 50 eggs/10 ml)	17	17	2.37	2540 [149.41 (57.52–241.31)]
*P-*value				0.2793
Fathers’ occupation				
Civil servants	249	43	5.99	571 [13.28 (8.42–18.14)]
Farmers	115	33	4.59	1523 [46.15 (−1.22–93.53)]
Other brown collar jobs	351	87	12.12	1989 [22.86 (11.95–33.78)]
Late (Dead)	2	0	0	0
Retiree	1	0	0	0
*P-*value			0.0814	0.9995
Mothers’ occupation				
Civil servants	76	7	0.97	110 [15.71 (−0.27–31.69)]
Farmers	2	0	0	0
Other brown collar jobs	390	96	13.37	1983 [20.66 (11.63–29.68)]
Housewives	250	60	8.36	1990 [33.17 (6.44–59.89)]
*P-*value			0.0231	1.0000
Total	718	163	22.7	4083 (25.05)

**Fig. 4:**
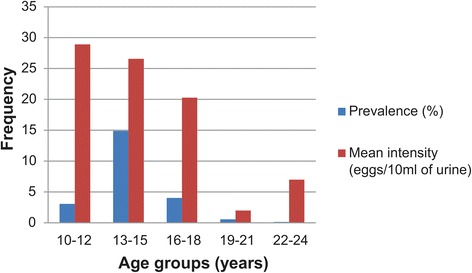
Age group-related prevalence and intensity with urogenital schistosomiasis in the study LGAs

The prevalence and mean egg count (intensity) was higher in males [19.5 % (*χ*^2^ = 77.065, *P* < 0.0001) and 28.7 eggs/10 ml of urine)] than females (3.2 % and 2.83 eggs/10 ml of urine). The highest prevalence (14.9 %) was recorded among children in the age group 13–15 years (*χ*^2^ = 7.857, *P* = 0.0197) compared to age groups 10–12, 16–18, 19–21 and 22–24 years with prevalence rates of 3.06 %, 4.04 %, 0.56 % and 0.14 %, respectively. However, as shown in Fig. 4, a seemingly highest mean intensity (28.91 eggs/10 ml of urine) was recorded for age group 10–12 years (*χ*^2^ = 13.880, *P* = 0.9978) compared to 13–15, 16–18, 19–21, and 22–24 years with mean intensities of 26.58, 20.28, 2 and 7 eggs/10 ml of urine, respectively (Table 4). While there was insignificant association (0.9694) and weak linear relationship (*r* = -0.0014) between age of respondents and *S. haematobium* egg counts, a statistically significant association (*P* < 0.0001) and weak linear relationship (*r* = -0.3414) was recorded between the sex of respondents and egg counts of *S. haematobium*.

Of the total number of respondents infected, 146 (89.57 %) fell into the light intensity category while 17 (10.43 %) belonged to the heavy intensity category. By location, 90 (55.21 %) and 73 (44.79 %) of the infected respondents came from Dutsin-Ma and Safana LGAs, respectively. Conversely, of 4083 *S. haematobium* eggs recovered, Dutsin-Ma recorded 2314 eggs (mean = 25.71 (8.05–43.37) eggs/10 ml of urine)] while Safana recorded 1769 eggs (mean = 24.23 (12.07–36.39) eggs/10 ml of urine]. Invariably, Dutsin-Ma recorded a higher prevalence of 12.53 % and mean intensity.

Highest prevalence [12.12 % (*χ*^2^ = 7.236; *P* = 0.0071)] was recorded among children whose fathers’ occupation belonged to the category of “other brown collar jobs” while the highest mean intensity [46.15 (-1.22–93.53) eggs/10 ml of urine)] of the infection was identified among children whose fathers’ jobs fell into the category of “farming”. As far as prevalence was concerned, the same scenario repeated for children whose mothers’ occupations belonged to the category of “other brown collar jobs” with a highest prevalence of [13.37 % (*χ*^2^ = 100.523; *P* = 0.0001)]. The highest mean intensity [33.17 (6.44–59.89) eggs/10 ml of urine] was identified among children whose mothers were housewives, followed by other brown collar jobs [20.66 (11.63–29.68)] with civil servants as the least [15.71 (−0.27–31.69)] eggs/10 ml of urine (Table 4).

In the bivariate analysis, sex (boys) [COR (95 % CI): 6.86 (4.28–10.99)] and age group 16–18 years [COR (95 % CI): 2.11 (1.25–3.56)] were significantly associated with genito-urinary schistosomiasis. Meanwhile, altitude [COR (95 % CI): 1.69 (1.18–2.45)] played a major role in the transmission of the infection unlike address [COR (95 % CI): 1.01 (0.71–1.44)] whose role was insignificant. In fathers’ occupational category, only farming [COR (95 % CI): 1.93 (1.14–3.24)] and other brown collar jobs [COR (95 % CI): 1.58 (1.05–2.39)] were found to be significantly associated with *S. haematobium* infection.

In mothers’ occupational category, only other brown collar job [COR (95 % CI): 3.22 (1.49–7.82)] and housewives [COR (95 % CI): 3.11 (1.41–7.68)] were found to be significantly associated with the disease (Table 5). In the multivariate logistics regression analysis, only age and sex had statistically significant association with genito-urinary schistosomiasis.

**Table 5:** Multivariate analysis of the variables associated with the frequency of genito-urinary schistosomiasis among respondents

Variables	Subtotal	Positive (%)	Negative (%)	COR (95 % CI)	AOR (95 % CI)
Sex					
Boys	401	140 (34.91)	261 (65.09)	6.84 (4.32–11.17)	7.25 (4.33–12.71)
Girls	317	23 (7.26)	294 (92.74)	1	1
*χ* ^*2*^ test				77.065	–
*P*-value				< 0.0001	–
Age (years)					
10–12	126	22 (17.46)	104 (82.54)	1	1
13–15	485	107 (22.06)	378 (77.94)	1.34 (0.81–2.26)	–
16–18	94	29 (30.85)	65 (69.15)	2.11 (1.11–4.01)	4.43 (2.62–7.49)
19–21	11	4 (36.36)	7 (63.64)	2.70 (0.64–10.08)	–
22–24	2	1 (50)	1 (50)	4.73 (0.12–185.89)	–
*χ* ^*2*^ test				0.642	–
*P-*value				0.4231	–
Address					
Dutsin-Ma	398	90 (22.61)	308 (77.39)	1	1
Safana	320	73 (22.81)	247 (77.19)	1.01 (0.71–1.44)	–
*χ* ^*2*^ test				0.004	–
*P-*value				0.9495	–
Altitude					
500*–*530 m	312	55 (17.63)	257 (82.37)	1.69 (1.18–2.45)	1.27 (0.87–1.88)
531–560 m	406	108 (26.61)	298 (73.39)	1	1
*χ* ^*2*^ test				8.083	–
*P*-value				0.0045	–
Fathers’ occupation					
Civil servants	249	43 (17.27)	206 (82.73)	1	1
Farmers	115	33 (28.69)	82 (71.3)	1.93 (1.14–3.24)	2.08 (1.2–3.59)
Other brown collar jobs	351	87 (24.79)	264 (75.21)	1.58 (1.05–2.39)	1.42 (0.92–2.23)
Late (Dead)	2	0 (0)	2 (100)	0	–
Retiree	1	0 (0)	1 (100)	0	–
*χ* ^*2*^ test				0.0001	
*P-*value				0.9921	–
Mothers’ occupation					
Civil servants	76	7 (9.21)	69 (90.79)	1	–
Farmers	2	0 (0)	2 (100)	0	–
Other brown collar jobs	390	96 (24.62)	294 (75.38)	3.22 (1.49–7.82)	–
Housewives	250	60 (24)	190 (76)	3.11 (1.41–7.68)	–
*χ* ^*2*^ test				6.359	–
*P-*value				0.0117	–
Grand total	718	163 (22.7)	555 (77.3)		

*Abbreviations*: *COR* Crude (unadjusted) odds ratio, *AOR* adjusted odds ratio (indicated only for values with *P* ≤ 0.05), *CI* Confidence interval

Boys [AOR (95 % CI): 7.3 (4.26–12.4)] were seven times more likely to be infected with the cercariae of *S. haematobium* compared to girls. Moreover, age group 16–18 years [AOR (95 % CI): 4.43 (2.62–7.49)] was about four times more likely to be infected compared to 10–12 years. Students whose fathers were farmers [AOR (95 % CI): 2.08 (1.2–3.59)] were twice more likely to be infected than those whose fathers were civil servants (Table 5).

## Discussion

### Prevalence of genito-urinary schistosomiasis

In this cross-sectional survey, the overall prevalence of genito-urinary schistosomiasis among junior high school students from the LGAs was 22.7 %. They were categorised as moderate risk (≥ 10 % but < 50 %) LGAs [15]. By logical deduction, water contact activities are expected to be higher in the middle belt and southern part of Nigeria compared to the North. This probably informed higher prevalences of 37.9 % [16], 43.7 % [17] and 55 % [2] obtained in Cross River, Ondo and Benue States, respectively. Nevertheless, the prevalence obtained from this study is higher compared to 2.07 % and 16.3 % reported in Jos [18] and Nasarawa [19], respectively, both being North Central States. However, it is low compared to those obtained in Sokoto (37.7, 38.3 and 60.8 %) [20–22] and 61.7 % obtained in Zamfara [23], all northwestern states of Nigeria. While a prevalence of 57.6 % was recorded in Fatick region of Senegal [10], 52.8 % was reported in north-eastern region of Zimbabwe [24]. Meanwhile, in highly endemic foci of Malawi, current data show that prevalence rates as high as 90–100 % have been recorded among children aged five and 15 years [25].

The prevalence rates obtained in this study are highly informative. These are a function of the interplay of various factors ranging from ecological to socio-economic which in turn took toll on the water contact activities of the study population. To corroborate this, in Katsina State, as at 2010, 0.2 and 6.4 % of the households used toilet on water and lacked toilet facility, respectively, while as at 2009, 23.6 % households had their toilets within a distance of 500 m. Moreover, as at 2009, mean annual rainfall of 473.8 mm and temperature range of 21.3–34.5 °C were recorded [7]. These factors limit the network of freshwater bodies and ensure that the few available become characteristically infested with *S. haematobium* larvae.

Furthermore, a higher prevalence of 19.5 % was recorded in boys compared to 3.2 % in girls after the study. Some researchers have previously documented higher prevalence rates in boys: (boys: 45.5 %; girls: 37.2 %) [26], (boys: 18.7 %; girls: 8.11 %) [19], (boys: 37 %; girls: 31.2 %) [27] and (boys: 8.2 %; girls: 5.2 %) [28]. However, the result of this survey contradicted the findings at Ebonyi (boys: 44.9 %; girls: 51.9 %) [29] and Ogun (boys: 57.1 %; girls: 59.2 %) [12] states in Nigeria. Another report of higher prevalence rate in females was given in Ogun State (boys: 22.4 %; girls: 23.2 %) [30] and Sudan (boys: 22 %; girls: 34 %) [31].

These results were actually anticipated due to the focal nature of the infection. The higher prevalence rate recorded among boys is suggestive of more water contact activities like swimming, playing with shallow water and fishing, in addition to the normal duty of fetching water and washing which are peculiar to both sexes [2, 20, 26].

Prevalence increased with age as shown by this survey. However, the highest prevalence of 14.9 % was recorded in the age group 13–15 years. This quite agrees with previous findings which reported high prevalences for 13–15 years [13, 27]; 11–15 years [2, 19]; 12–16 years [32]; and 13–16 years [33].

In this study, respondents who relied on unwholesome water sources recorded higher values of prevalence. Meanwhile, previous findings have shown that access to wholesome water sources was associated with significantly lower odds of infection with *S. haematobium* [34]. Lack of pure and potable water supply has been identified as a major factor in the spread of the disease [19]. This is further attested to by the report from Katsina State as at 2010 where 4.6 % of households had access to treated pipe-borne water while 9.6 % depended on rivers [7].

Finally, children whose parental occupation belonged to the category of brown collar jobs (farming and other brown collar jobs) suffered more from the menace of the disease. Respondents with fathers in this group had a prevalence of 16.71 % while those with mothers in similar group had a prevalence of 13.37 %. In Ethiopia, a higher prevalence (56 %) was reported among subjects whose fathers were farmers [11].

### Intensity of genito-urinary schistosomiasis

In this survey, boys recorded a higher mean intensity (28.7 eggs/10 ml of urine) compared to girls (2.83 eggs/10 ml of urine). This is consistent with previous reports: (boys: 9.36 eggs/10 ml; girls: 7.81 eggs/10 ml) [11], (boys: 32 eggs/10 ml; girls: 21.5 eggs/10 ml) [18] and (boys: 45.45 eggs/10 ml; girls: 36.58 eggs/10 ml) [22]. This pattern of intensity suggests a higher frequency of contact with infested water bodies by boys through swimming, fishing, playing in shallow water and fetching water for construction work.

Moreover, mean intensity decreased with age. This could be explained by reduction in the frequency of water contact activities among older students as a result of maturity and acquisition of knowledge about the disease by formal education. In a study conducted in Nigeria, similar result was obtained [35]. Age group 10–12 years recorded the highest mean intensity (28.91 eggs/10 ml of urine) while age group 13–15 years, the second highest, recorded 26.58 eggs/10 ml of urine. In surveys conducted at Plateau and Ebonyi States of Nigeria, 10–14 years was reported as the age group with the highest intensity [18, 36].

A dramatic twist occurred in the pattern of mean intensity obtained in this study with respondents who adhered strictly to the usage of tap, well, borehole and sachet water sources recording the highest mean intensity of 55.78 eggs/10 ml of urine. Noteworthy was the fact that all the respondents had a previous experience of swimming in *S. haematobium* cercariae-infested water bodies. Respondents who, however, combined the habits of swimming with playing in shallow water bodies recorded the highest mean intensity (35.9 eggs/10 ml of urine) in their category, with swimmers as the second highest (22.43 eggs/10 ml of urine). Previous studies have confirmed the possibility of *S. haematobium* infection in those who depended on wholesome water sources (for domestic use) subject to contact with unwholesome water sources for recreation. To corroborate this, mean intensity of 9.5 eggs/10 ml of urine was reported in interviewees who relied on borehole water for consumption [35]. The high rate of the infection in some villages supplied with tap water in Senegal was attributed to similar factor as well as the proximity to ponds, the lack of health education and poor hygiene [10].

A higher mean intensity (31.69 eggs/10 ml of urine) was recorded in Dutsin-Ma LGA compared to Safana LGA (19.66 eggs/10 ml of urine). This might be attributed to the location of Zobe Dam in the former, suggesting a stronger network of rivers, ponds and streams which is a key factor in the epidemiology of genito-urinary schistosomiasis.

As far as infection category was concerned, 17 (2.37 %) respondents of the whole 718 surveyed accounted for the heavy mean intensity of 149.41 eggs/10 ml of urine recorded compared to 146 (20.33 %) subjects who accounted for a light mean intensity of 10.57 eggs/10 ml of urine. Previous studies have reported that majority of infected subjects belonged to the “light infection” category [21, 23, 26]. Interviewees whose fathers were farmers recorded the highest mean intensity (46.21 eggs/10 ml of urine) while those whose fathers’ jobs belonged to the “other brown collar jobs” category served as the second highest (22.84 eggs/10 ml of urine). Again, this result agrees to the report of a survey conducted in Zamfara State, Nigeria [21]. Noteworthy is the fact that there is a dearth of literature addressing the intensity of infection among school children based on their parents’ occupations. Conversely, in respondents whose mothers were civil servants, the highest mean intensity (39.33 eggs/10 ml of urine) was recorded while those whose mothers were housewives served as the second highest (36.23 eggs/10 ml of urine). It was previously reported that the disease was common among children whose mothers or fathers were civil servants [37]. However, no figure was given as regards the intensity of infection. Theoretically, such children were not expected to be infected. However, with the common knowledge that some civil servants are illiterates as well as potential brown collar jobs seekers, this result is justifiable.

### Relationship between genito-urinary schistosomiasis and some variables

In this study, fathers’ occupation (farming), sex and age were significantly associated with the disease. Respondents with farming as fathers’ occupation were twice [AOR (95 % CI): 2.08 (1.2–3.59)] more likely to be infected compared to those whose fathers were civil servants. This is consistent with the findings of researchers from Ethiopia [11] and Sudan [38]. An average civil servant is more likely to be literate than a peasant farmer. Where school children participate in farming activities with their illiterate and uninformed fathers, they suffered a higher risk of being infected.

Boys were found to be seven times [AOR (95 % CI): 7.3 (4.26–12.4)] more likely to be infected than girls. This is indeed rare in the literature. It could be explained by their involvement, to a very large extent, in various water contact activities than girls.

Cultural practices, coupled with the need to eke out a living as a result of the prevailing poverty may behaviourally predispose males to more contacts with open and potentially infested water bodies. Previous studies have documented males as having higher odds of being infected compared to females [11, 33, 39].

## Conclusions

In this study, Dutsin-Ma and Safana in Katsina State, Nigeria, were classified as moderate-risk LGAs for genito-urinary schistosomiasis. Age, sex, fathers’ and mothers’ occupations were identified as the determining epidemiological factors in the prevalence of the disease. Sustainable chemotherapeutic intervention with Praziquantel® is highly recommended to reduce its prevalence below the threshold of public health significance. To compliment this, a good network of treated pipe-borne water, health education and waste disposal facilities should be urgently but adequately provided.

## Abbreviations

AOR, Adjusted odds ratio; CI, Confidence interval; CL, Confidence level; COR, Crude odds ratio; LGAs, Local government areas; LGEA, Local government education authority area; OR, Odds ratio; SD, Standard deviation; SPSS, Statistical package for social sciences
